# Effect of the “universal test and treat” policy on the characteristics of persons registering for HIV care and initiating antiretroviral therapy in Uganda

**DOI:** 10.3389/fpubh.2023.1187274

**Published:** 2023-06-09

**Authors:** Levicatus Mugenyi, Christian H. Hansen, Philippe Mayaud, Janet Seeley, Robert Newton, Mastula Nanfuka, Andrew Abaasa, Kenneth Mugisha, Michael Etukoit, Pontiano Kaleebu, Eugene Ruzagira

**Affiliations:** ^1^MRC/UVRI and LSHTM Uganda Research Unit, Entebbe, Uganda; ^2^London School of Hygiene & Tropical Medicine, London, United Kingdom; ^3^The AIDS Support Organization, Kampala, Uganda

**Keywords:** universal test and treat, HIV/AIDS, antiretroviral therapy, TASO, Uganda

## Abstract

We examined the effect of the Universal Test and Treat (UTT) policy on the characteristics of people living with HIV (PLHIV) at enrolment in HIV care and initiation of antiretroviral therapy (ART) in Uganda using data from 11 nationally representative clinics of The AIDS Support Organisation (TASO). We created two retrospective PLHIV cohorts: pre-UTT (2004–2016), where ART initiation was conditional on CD4 cell count and UTT (2017–2022), where ART was initiated regardless of World Health Organisation (WHO) clinical stage or CD4 cell count. We used a two-sample test of proportions and Wilcoxon rank-sum test to compare proportions and medians, respectively, between the cohorts. A total of 244,693 PLHIV were enrolled at the clinics [pre-UTT, 210,251 (85.9%); UTT, 34,442 (14.1%)]. Compared to the pre-UTT cohort, the UTT cohort had higher proportions of PLHIV that were male (*p* < 0.001), aged 18–29 years (p < 0.001), aged >69 years, never married (p < 0.001), and educated to primary (p < 0.001) and post-primary (p < 0.001) school level at enrolment in HIV care and ART initiation. Overall, 97.9% of UTT PLHIV initiated ART compared to 45.2% under pre-UTT. The median time from enrolment in HIV care to ART initiation decreased from 301 [interquartile range (IQR): 58–878] pre-UTT to 0 (IQR: 0–0) under UTT. The median CD4 count at ART initiation increased from 254 cells/μL pre-UTT to 482 cells/μL under UTT (*p* < 0.001). Compared to the pre-UTT cohort, the UTT cohort had higher proportions of PLHIV with a CD4 count >500 cells/μL (47.3% vs. 13.2%, p < 0.001) and WHO stage 1 (31.7% vs. 4.5%, p < 0.001) at ART initiation. Adoption of the UTT policy in Uganda was successful in enrolling previously unreached individuals, such as men and younger and older adults, as well as those with less advanced HIV disease. Future research will investigate the effect of UTT on long-term outcomes such as retention in care, HIV viral suppression, morbidity, and mortality.

## 1. Introduction

In 2015, the World Health Organization (WHO) recommended that antiretroviral therapy (ART) should be initiated among all people living with HIV (PLHIV) regardless of WHO clinical stage and at any CD4 cell count, the so-called-Universal Test and Treat (UTT) policy (1). Uganda rolled out the UTT policy in 2017 (2). As UTT is implemented, it is expected that the profile of persons enrolling into HIV care and starting ART under the new system will be different from that of individuals who enrolled in care under previous, more conditional guidelines, where ART initiation was conditioned on CD4 counts dropping below certain threshold, i.e., <200 cells/μL from 2004 to 2010 (3), ≤350 cells/μL from 2010 to 2013 (4), and ≤ 500 cells/μL from 2013 to 2015 (5). Many of these people, unlike those initiating ART under earlier policies, are expected to be asymptomatic (6), and to include sub-populations that have historically been underrepresented in HIV care programs, e.g., men and young adults (7–9). Successful engagement of these sub-populations in HIV care is critical to achieving the goal of UTT, i.e., high levels of viral suppression and consequently reduced HIV-related morbidity and mortality as well as reduced risk of HIV transmission (10–12). However, given the relatively recent rollout of the UTT policy at the country level, there is little evidence to date of the policy’s real-world effect on these groups regarding engagement in HIV care in Uganda.

In the current study, we used routinely collected data from 11 clinics of The AIDS Support Organisation (TASO) to assess the impact of the UTT policy on the characteristics of the population enrolling in HIV care in Uganda.

## 2. Materials and methods

### 2.1. Setting and study population

TASO was founded in 1987 and is the oldest and largest community-based HIV care provider in Africa. Over the past 35 years, TASO has provided HIV prevention, care, and treatment services to more than 200,000 PLHIV through its clinics located across Uganda (13). A substantial number of PLHIV in Uganda receive care from TASO. For instance, of the estimated 1,200,000 Ugandan PLHIV currently receiving ART (14), 79,261 (6.6%) attend TASO clinics.

TASO began providing ART in 2004, following the various national threshold recommendations over time, treating individuals for whom CD4 counts were < 200 cells/μL (2004–2008), <250 cells/μL (2009–2011), <350 cells/μL (2012–2013), and < 500 cells/μL (2014–2016) and in 2017, adopting the UTT policy across all its clinics. Other services provided by TASO include HIV counselling and testing (HCT), clinical care, and clinical laboratory services including CD4 cell count and HIV viral load testing. Each person who registers with TASO is assigned a unique clinic registration number and their data (demographics, clinical, laboratory, etc.) are entered in a standardized database.

For the current study, we created two retrospective cohorts of all PLHIV registered into HIV care at the TASO clinics between 2004 and 2022. The first cohort (pre-UTT) included PLHIV registered during the period when ART initiation was conditional on CD4 cell count (2004–2016). The second cohort included PLHIV registered after implementation of the UTT policy (2017–2022).

### 2.2. Statistical analysis

Data variables both at enrolment and ART initiation included age in years, sex, marital status, level of education, and employment status. Additional data variables at ART initiation (not at enrolment) included CD4 cell count, WHO clinical stage, and date of ART initiation. We used descriptive statistics to compare characteristics of PLHIV in the two study cohorts at registration into TASO clinics and at time of ART initiation. We used a two-sample test of proportions to compare proportions and a Wilcoxon rank-sum test to compare medians across the two study cohorts. We excluded missing data from the analysis.

### 2.3. Ethical considerations

The TASO Institutional Review Board granted ethical approval for the study and waived the requirement to obtain informed consent as the study used anonymized secondary clinical data (TASOREC/096/19-UG-REC-009).

## 3. Results

### 3.1. Characteristics at enrolment in HIV care: Pre-UTT vs. UTT cohorts

**Figure 1:**
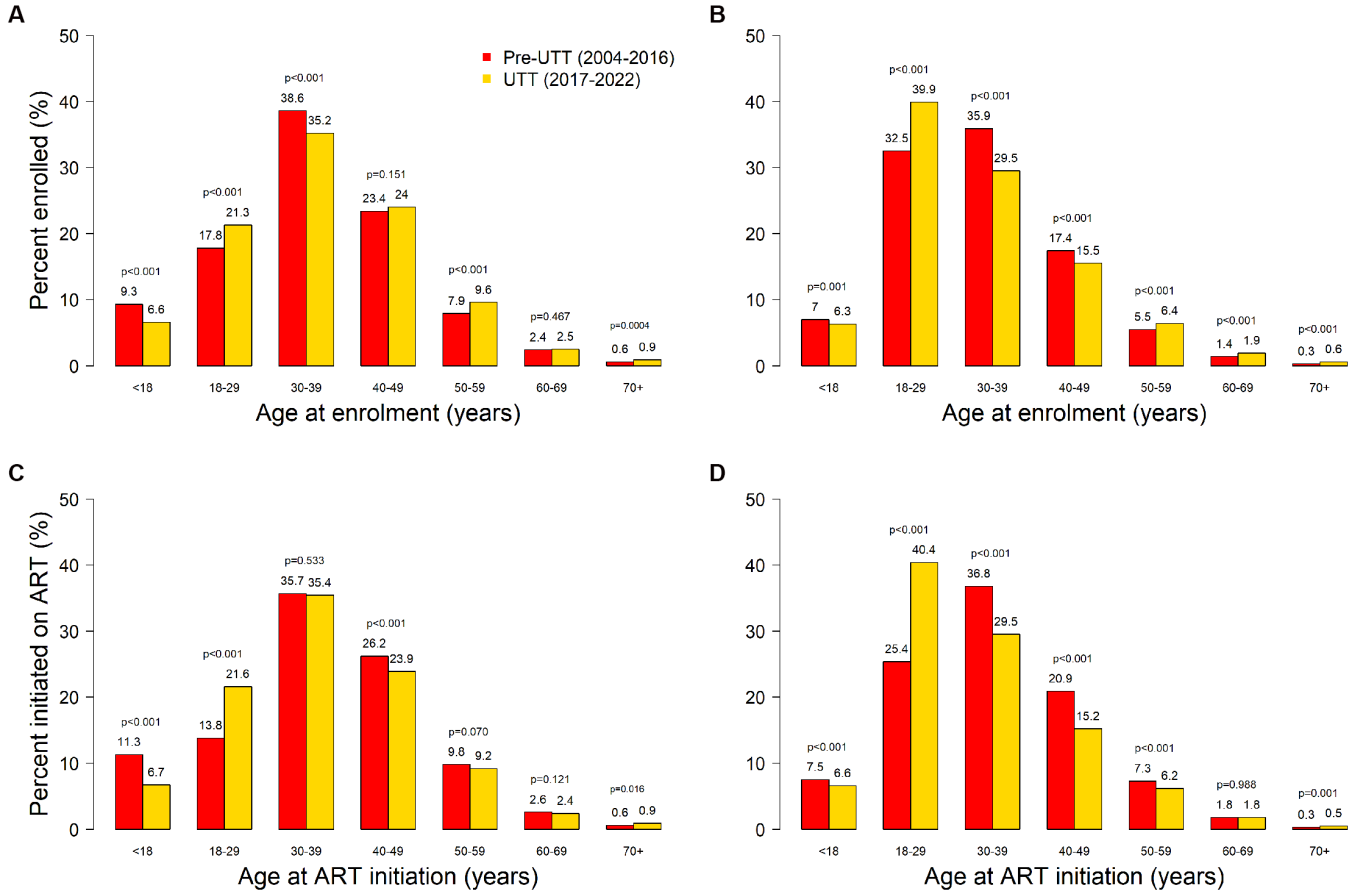
Sex-age distribution of PLHIV enrolling in HIV care among males (panel **A**) and females (panel **B**); and initiating ART among males (panel **C**) and females (panel **D**) in pre-UTT and UTT cohorts.

A total of 244,693 (female, 63.1%) PLHIV were enrolled across the TASO clinics between 2004 and 2022: 210,251 (85.9%) in the pre-UTT cohort and 34,442 (14.1%) in the UTT cohort. Baseline characteristics of PLHIV at enrolment in care by cohort are presented in Table 1. There was a greater proportion of men in the UTT cohort compared to the pre-UTT cohort (40.4% vs. 36.3%, *p* < 0.001). At enrolment, the UTT cohort was about one year younger than the pre-UTT cohort [median age 33.0 years (interquartile range [IQR]: 25.9–41.7) vs. 33.7 years (IQR: 27.2–40.8), p < 0.001). However, the proportions of PLHIV within age groups varied between cohorts. Compared to the pre-UTT cohort, the UTT cohort had a higher proportion of PLHIV in the 18-29-year (32.4% vs. 27.2%, *p* < 0.001), 50-59-year (7.7% vs. 6.4%, p < 0.001), 60-69-year (2.1% vs. 1.7%, p < 0.001) and > 69-year (0.7% vs. 0.4%, p < 0.001) age groups. Conversely, the proportions of PLHIV in the other age groups were lower in the UTT cohort compared to the pre-UTT cohort. This trend was largely similar in men and women (Figure 1).

**Table 1:** Baseline characteristics of people living with HIV at enrolment in HIV care and ART initiation in the pre-and during universal test and treat periods.

	**Enrolment into HIV care**				**ART initiation**			
**Characteristics**	**Pre-universal test and treat period (2004–2016) (N = 210,251)**	**Universal test and treat period (2017–2022) (N = 34,442)**	***p*-value** *	**All (2004–2022) (N = 244,693)**	**Pre-universal test and treat period (2004–2016) (N = 95,027)**	**Universal test and treat period (2017–2022) (N = 33,722)**	**P-value** *	**All (2004–2022) (N = 128,749)**
				
	**n (%)**	**n (%)**		**n (%)**	**n (%)**	**n (%)**		**n (%)**
**Sex**	**N = 210,251**	**N = 34,442**		**N = 244,693**	**N = 95,027**	**N = 33,722**		**N = 128,749**
Female	133,933 (63.7)	20,546 (59.7)	<0.001	154,479 (63.1)	61,680 (64.9)	20,123 (59.7)	<0.001	81,803 (63.5)
Male	76,318 (36.3)	13,896 (40.4)	<0.001	90,214 (36.9)	33,347 (35.1)	13,599 (40.3)	<0.001	46,946 (36.5)
**Age in years**	**N = 210,163**	**N = 34,433**		**N = 244,596**	**N = 90,345**	**N = 19,112**		**N = 109,457**
<18	16,460 (7.8)	2,215 (6.4)	<0.001	18,675 (7.6)	8,407 (8.9)	2,226 (6.6)	<0.001	10,633 (8.3)
18–29	57,098 (27.2)	11,151 (32.4)	<0.001	68,249 (27.9)	20,255 (21.3)	11,055 (32.8)	<0.001	31,310 (24.3)
30–39	77,494 (36.9)	10,942 (31.8)	<0.001	88,436 (36.2)	34,610 (36.4)	10,745 (31.9)	<0.001	45,355 (35.2)
40–49	41,203 (19.6)	6,523 (18.9)	0.004	47,726 (19.5)	21,614 (22.8)	6,303 (18.7)	<0.001	27,917 (21.7)
50–59	13,445 (6.4)	2,640 (7.7)	<0.001	16,085 (6.6)	7,763 (8.2)	2,501 (7.4)	<0.001	10,264 (8.0)
60–69	3,631 (1.7)	726 (2.1)	<0.001	4,357 (1.8)	1,955 (2.1)	675 (2.0)	0.535	2,630 (2.0)
>69	832 (0.4)	236 (0.7)	<0.001	1,068 (0.4)	422 (0.4)	216 (0.6)	<0.001	638 (0.5)
Median age (IQR)	33.7 (27.2, 40.8)	33.0 (25.9, 41.7)	<0.001	33.6 (27.1, 40.9)	35.3 (28.3, 42.7)	32.9 (25.7,41.3)	<0.001	34.7 (27.6, 42.4)
**Marital Status**	**N = 210,251**	**N = 34,442**		**N = 244,693**	**N = 95,027**	**N = 33,722**		**N = 128,749**
Currently married	100,226 (47.7)	16,973 (49.3)	<0.001	117,199 (47.9)	46,680 (49.1)	16,604 (49.2)	0.717	63,284 (49.2)
Separated/divorced	42,105 (20.0)	7,899 (22.9)	<0.001	50,004 (20.4)	18,567 (19.5)	7,711 (22.9)	<0.001	26,278 (20.4)
Widowed	40,627 (19.3)	2,133 (6.2)	<0.001	42,760 (17.5)	16,153 (17.0)	2,083 (6.2)	<0.001	18,236 (14.2)
Never married	13,600 (6.5)	4,096 (11.9)	<0.001	17,696 (7.2)	6,385 (6.7)	4,023 (11.9)	<0.001	10,408 (8.1)
Minor^§^	6,170 (2.9)	1,340 (3.9)	<0.001	7,510 (3.1)	4,109 (4.3)	1,306 (3.9)	<0.001	5,415 (4.2)
Not reported	7,523 (3.6)	2,001 (5.8)	<0.001	9,524 (3.9)	3,133 (3.3)	1,995 (5.9)	<0.001	5,128 (4.0)
**Highest level of education**	**N = 209,456**	**N = 31,400**		**N = 240,856**	**N = 94,759**	**N = 30,690**		**N = 125,449**
None/pre-primary	43,564 (20.8)	4,464 (14.6)	<0.001	48,028 (19.9)	18,955 (20.0)	4,345 (14.2)	<0.001	23,300 (18.6)
Primary	118,274 (56.5)	18,063 (57.5)	<0.001	136,337 (56.6)	52,517 (55.4)	17,679 (57.6)	<0.001	70,196 (56.0)
Post-primary	46,422 (22.2)	8,802 (28.0)	<0.001	55,224 (22.9)	22,738 (24.0)	8,598 (28.0)	<0.001	31,336 (25.0)
Other	1,196 (0.6)	71 (0.2)	<0.001	1,267 (0.5)	549 (0.6)	68 (0.2)	<0.001	617 (0.5)
**Employment**	**N = 202,533**	**N = 30,891**		**N = 233,424**	**N = 91,698**	**N = 30,206**		**N = 121,904**
Unemployed	40,323 (19.9)	5,401 (17.5)	<0.001	45,724 (19.6)	16,369 (17.9)	5,299 (17.5)	0.224	21,668 (17.8)
Employed^‡^	146,496 (72.3)	23,737 (76.8)	<0.001	170,233 (72.9)	66,855 (72.9)	23,197 (76.8)	<0.001	90,052 (73.9)
Student	2,939 (1.5)	97 (0.3)	<0.001	3,036 (1.3)	1,499 (1.6)	95 (0.3)	<0.001	1,594 (1.3)
Minor^#^	12,775 (6.3)	1,656 (5.4)	<0.001	14,431 (6.2)	6,975 (7.6)	1,615 (5.4)	<0.001	8,590 (7.1)
**CD4 count (cells/μL)**	-	-	-	-	**N = 43,528**	**N = 10,446**		**N = 53,974**
<200	-	-	-	-	16,048 (38.9)	2,151 (20.6)	<0.001	18,199 (33.7)
200–500	-	-	-	-	21,719 (49.9)	3,350 (32.1)	<0.001	25,069 (46.5)
>500	-	-	-	-	5,761 (13.2)	4,945 (47.3)	<0.001	10,706 (19.8)
*Median count (IQR)*	-	-	-	-	254 (146, 398)	482 (247, 751)	<0.001	2,782 (155, 457)
**WHO stage**					**N = 48,060**	**N = 26,549**		**N = 74,609**
Stage 1	-	-	-	-	2,167 (4.5)	8,410 (31.7)	<0.001	10,577 (14.2)
Stage 2	-	-	-	-	37,470 (78.0)	16,820 (63.4)	<0.001	54,290 (72.8)
Stage 3	-	-	-	-	6,941 (14.4)	970 (3.7)	<0.001	7,911 (10.6)
Stage 4	-	-	-	-	1,482 (3.1)	349 (1.3)	<0.001	1,831 (2.5)
**Time to ART initiation (Days)** **					**N = 93,939**	**N = 31,410**		**N = 125,349**
Median days (IQR)	-	-	-	-	301 (58, 878)	0 (0, 0)	<0.001	109 (0, 624)

* *Two-sample test of proportions or Wilcoxon rank-sum test for medians.*

*IQR: Interquartile range*

§ *Includes persons ≤ 18 years who were not currently married and had never been married.*

‡ *Includes persons in formal employment, self-employment (small-scale farmers, businesspersons, etc.), and hired/casual labourers.*

# *Includes persons ≤ 18 years who were neither employed nor in school.*

** *Excludes persons whose date of ART initiation was missing or preceded registration for TASO care.*

The proportion of widowed PLHIV was lower in the UTT cohort compared to the pre-UTT cohort (6.2% vs. 19.3%, *p* < 0.001). The proportions of PLHIV reporting other marital status categories were higher in the UTT cohort compared to the pre-UTT cohort (currently married, 49.3% vs. 47.7%, p < 0.001; separated/divorced, 22.9% vs. 20.0%, p < 0.001; never married, 11.9% vs. 6.5%, p < 0.001).).

The proportion of PLHIV with primary education was higher in the UTT cohort than in the pre-UTT cohort (57.5% vs. 56.5%, *p* < 0.001). Also, the proportion with post-primary education was higher in the UTT cohort than in the pre-UTT cohort (28.0% vs. 22.2%, *p* < 0.001). On the other hand, the proportion of PLHIV with no education/pre-primary education was lower in the UTT cohort compared to the pre-UTT cohort (14.6% vs. 20.8%, *p* < 0.001).

The proportion of employed PLHIV was higher in UTT cohort compared to the pre-UTT cohort (76.8% vs. 72.3%, *p* < 0.001).

### 3.2. Characteristics at ART initiation: Pre-UTT vs. UTT cohorts

Of the 210,251 PLHIV in the pre-UTT period, 95,027 (45.2%) initiated ART. Of the 115,224 PLHIV who did not initiate ART, 96,708 (83.9%) were lost to follow-up, 10,612 (9.2%) transferred to another HIV care provider, 4,750 (4.1%) were known to have died, and 3,154 (2.7%) were still receiving care at TASO by the end of the year 2016 (just before UTT). Among the 34,442 PLHIV in the UTT cohort, 33,722 (97.9%) had initiated ART. Of the 720 PLHIV who had not initiated ART, 129 (17.9%) were still receiving care at TASO, 535 (74.3%) were lost to follow-up, 34 (4.7%) transferred to another HIV care provider, and 22 (3.1%) had died by the end of year 2022. The median time from enrolment in HIV care to ART initiation was shorter in the UTT cohort compared to the pre-UTT cohort (0 days vs. 301 days, *p* < 0.001).

Characteristics of PLHIV at ART initiation are presented in Table 1. A higher proportion of male PLHIV initiated ART in the UTT cohort compared to the pre-UTT cohort (40.3% vs. 35.1%, *p* < 0.001). The median age at ART initiation was lower in the UTT cohort compared to the pre-UTT cohort (Overall 32.9 years vs. 35.3 years, *p* < 0.001; Women 30.6 years vs. 34.2 years, p < 0.001; Men 35.9 years vs. 37.0 years, *p* < 0.001).

Compared to the pre-UTT cohort, the UTT cohort had a higher proportion of PLHIV initiating ART in the 18-29-year (32.8% vs. 21.3%, *p* < 0.001) and > 69-year (0.6% vs. 0.4%, p < 0.001) age groups. Conversely, the proportions of PLHIV in the other age groups were either lower in the UTT cohort compared to the pre-UTT cohort (<18 years, 6.6% vs. 8.9%, *p* < 0.001; 30–39 years, 31.9% vs. 36.4%, *p* < 0.001; 40–49 years, 18.7% vs. 22.8%, *p* < 0.001; 50–59 years, 7.4% vs. 8.2%, p < 0.001) or similar between the cohorts (60–69 years, 2.0% vs. 2.1%, *p* = 0.535).

The proportion of widowed PLHIV was lower in the UTT cohort compared to the pre-UTT cohort (6.2% vs. 17.0%, *p* < 0.001). The proportions of PLHIV reporting other marital status categories were higher in the UTT cohort compared to the pre-UTT cohort (Separated/divorced, 22.9% vs. 19.5%, *p* < 0.001; Never married, 11.9% vs. 6.7%, p < 0.001). The proportions for the currently married were similar between the UTT and pre-UTT cohrts (49.2% vs. 49.1%, *p* = 0.717).

The proportion of PLHIV with primary education was higher in the UTT cohort than in the pre-UTT cohort (57.6% vs. 55.4%, p < 0.001). Similarly, the proportion of PLHIV with post-primary education was higher in the UTT cohort compared to the pre-UTT cohort (28.0% vs. 24.0%, p < 0.001). On the other hand, the proportion of PLHIV with no education/pre-primary education was lower in the UTT cohort compared to the pre-UTT cohort (14.2% vs. 20.0%, p < 0.001).

The proportion of employed PLHIV was higher in the UTT cohort compared to the pre-UTT cohort (76.8% vs. 72.9%, p < 0.001).

The median CD4 count at ART initiation was higher in the UTT cohort compared to the pre-UTT cohort (482 cells/μL vs. 254 cells/μL, p < 0.001). The proportion of PLHIV with low CD4 counts at ART initiation was lower in the UTT cohort compared to the pre-UTT cohort (<200 cells/μL, 20.6% vs. 38.9%, p < 0.001; 200–500 cells/μL, 32.1% vs. 49.9%, p < 0.001). More PLHIV had a CD4 count >500 cells/μL in the UTT cohort compared to the pre-UTT cohort (47.3% vs. 13.2%, p < 0.001).

Compared to the pre-UTT cohort, the UTT cohort had a higher proportion of PLHIV in WHO stage 1 (31.7% vs. 4.5%, p < 0.001) and lower proportions of PLHIV in WHO stage 2 (63.4% vs. 78.0%, p < 0.001), WHO stage 3 (3.7% vs. 14.4%, p < 0.001) and WHO stage 4 (1.3% vs. 3.1%, p < 0.001).

## 4. Discussion

Our results show substantial changes in the sociodemographic, clinical, and immunological profiles of persons enrolling for HIV care and initiating ART after implementation of the UTT policy in Uganda. With regard to sociodemographic characteristics, the study found that UTT has been relatively successful at reaching more men, young adults, persons with high levels of formal education and those in employment, population groups that have been historically difficult to engage in HIV care (15–17). This is important since adequate treatment coverage for all demographic groups is necessary to fully realise the population-level benefits of UTT (18).

Overall, more women than men enrolled in HIV care and initiated ART in the pre-UTT and UTT cohorts. There was however a significant increase in the proportion of men who enrolled in HIV care and initiated ART under UTT. This is probably because UTT has eliminated barriers such as time spent travelling to and waiting at health facilities, e.g., for CD4 eligibility screening, before ART initiation that have been previously cited for men’s low utilisation of HIV care services. Other studies have reported improved rates of ART initiation among men following adoption of UTT but also noted that men remain at greater risk of not initiating ART rapidly (19). Women in sub-Saharan Africa are disproportionately affected by HIV. For example, one in every five new infections occurs among adolescent girls and young women despite this group making up only 10% of the population (20). Nonetheless, effective control of the HIV epidemic in this region where HIV transmission is largely heterosexual will require engagement of men (11). If men are successfully engaged in HIV care and virally suppressed, they will be less likely to transmit HIV to their female partners. In turn, if fewer women get HIV from their male partners, fewer children will be at risk of acquiring HIV from their mothers (21). Hence it is critical to continuously monitor and seek ways to address sex disparities in engagement in HIV care.

According to the Uganda population-based HIV impact assessment survey (UPHIA) 2016–2017, only 40% of PLHIV in the 20-29-year age group were receiving ART versus 60 to 80% among PLHIV in older age groups (17). It is therefore notable that we found a large increase in the proportion of 18-29-year-old PLHIV enrolling in HIV care and initiating ART under UTT. Since younger persons tend to be more sexually active than their older counterparts, it can be expected that increased engagement of this population in HIV care will result in a substantial impact on HIV incidence (15). The increase in the proportions of PLHIV older than 69 years in the UTT cohort points to the possibility of recent infection in this population and may be an indication that older persons in this setting are also vulnerable to HIV infection. Indeed, recent studies elsewhere in sub-Saharan Africa have reported high HIV incidence among older persons (22). We observed a decrease in the proportion of 30-49-year-old PLHIV enrolling in HIV care and initiating ART in the UTT cohort. This is probably because these individuals are likely to have had HIV for longer and consequently to have encountered the healthcare system earlier than younger PLHIV. The observed decrease in the proportion of PLHIV younger than 18 years under UTT is not attributed to UTT but is probably due to the reduced number of children newly infected with HIV due to a general trend observed because of the increased availability of ART. According to UNAIDS, the number of children (aged 0–14 years) newly infected with HIV in Uganda decreased from over 20,000 in 2010 to 6,000 in 2021 owing to the increasingly effective strategies for the elimination of mother-to-child transmission (23, 24).

Under previous treatment guidelines, inability to take time off work for clinic appointments was a common barrier to engagement in HIV care among employed individuals and those with a high education level (25, 26). The observed increase in the proportion of PLHIV with post-primary education and those in employment under UTT can probably be attributed to the elimination of time demanding ART eligibility determination processes such as determination of CD4 cell counts and the associated multiple clinic visits.

UTT led to increases in the proportions of PLHIV enrolling in HIV care and starting ART in all marital status categories except for the widowed. We found a greater than 60% decrease in the proportion of widowed PLHIV enrolling in HIV care and starting ART in the UTT period. This finding may be attributed to decreased levels of HIV/AIDS-related widowhood. Previous studies have shown that HIV/AIDS-related deaths are common cause of widowhood in sub-Saharan Africa (27, 28) with the highest risk of widowhood among sero-concordant-positive couples (29). Due to increasing availability of ART in Uganda over the years, AIDS-related deaths have declined substantially. For instance, 51,000 deaths were attributed to HIV/AIDS in 2010 compared to 17,000 in 2021, a reduction of 67% (30). Hence, it is probable that there has been a corresponding decrease in the population of HIV-positive widows. It is also possible that most of the surviving widows had already been enrolled in HIV care during the pre-UTT period. This possibility is supported by results from a national survey that ended a few months into the UTT period, which showed that nearly 80% of widowed PLHIV were receiving ART versus 40 to 60% among their non-widowed counterparts (17).

We found that PLHIV in UTT had less advanced disease at enrolment in HIV care and ART initiation than their pre-UTT counterparts. There was a substantial increase in the proportion of PLHIV starting ART at WHO stage 1 as well as those with a CD4 count >500 cells/μL under UTT. Overall, the median CD4 count at ART initiation increased from 254 cells/μL pre-UTT to 482 cells/μL under UTT, an increase of 89.8%. These results are encouraging given that the high HIV mortality and incidence observed in sub-Saharan Africa have been largely attributed to low median CD4 counts at enrolment in HIV care and ART initiation (9, 15).

We found that 97.9% of PLHIV who enrolled in HIV care after implementation of UTT initiated ART compared to 45.2% in the pre-UTT period. Furthermore, there was a marked reduction in time between enrolment in HIV care to ART initiation following implementation of the UTT policy, from a median of 301 days to 0 days. Other studies have reported similarly rapid rates of ART initiation following adoption of UTT in Uganda (19).

The strengths of this study include use of routinely collected nationally representative data from real-world clinic settings and the large sample size. Hence the findings are likely to be generalisable to the whole country. Study limitations include the possibility that not all observed differences between the cohorts can be attributed to the introduction of UTT owing to the before-and-after design. For instance, as explained above, the decrease in the proportion of widowed PLHIV in the UTT period may be due to other factors such as reduced HIV/AIDS-related mortality over time. Another possible explanation for the study findings may be relative changes in the incidence of HIV infection in the different population groups between the cohort periods. However, available data on new HIV infections in Uganda suggest that this is unlikely. For example, whereas the proportion of men enrolling in HIV care and starting ART increased under UTT, the proportion of new HIV infections contributed by men decreased from 41% in 2010 to 34% in 2021 (30). Another limitation is the large amount of missing data on CD4 cell counts and WHO clinical stages. Finally, the large sample size may have made it possible to detect small but unimportant differences between the two cohorts.

In conclusion, these findings show that adoption of the UTT policy in Uganda was successful in reaching men and young adults as well as PLHIV with less advanced HIV disease with ART. Future research will investigate the effect of UTT on long-term outcomes such as retention in care, HIV viral suppression, morbidity, and mortality.

## Data availability statement

The raw data supporting the conclusions of this article will be made available by the authors, without undue reservation.

## Ethics statement

The studies involving human participants were reviewed and approved by TASO Institutional Review Board. Written informed consent from the participants’ legal guardian/next of kin was not required to participate in this study in accordance with the national legislation and the institutional requirements.

## Author contributions

LM: managed data, analysed data, interpreted results, and drafted and edited the manuscript. CH: supervised LM, interpreted the results, and edited the manuscript. PM: conceived the idea, interpreted the results, and edited the manuscript. JS, RN, and AA: interpreted the results and edited the manuscript. MN and KM: data collection, interpreted the results, and edited the manuscript. ME and PK: supervisory and funds sourcing. ER: conceived the idea, interpreted the results, drafted, and edited the manuscript. All authors contributed to the article and approved the submitted version.

## Funding

This study was jointly funded by the UK Medical Research Council (MRC) and the UK Department for International Development (DFID) under the MRC/DFID Concordat agreement and is also part of the EDCTP2 program supported by the European Union.

## Conflict of interest

The authors declare that the research was conducted in the absence of any commercial or financial relationships that could be construed as a potential conflict of interest.

## Publisher’s note

All claims expressed in this article are solely those of the authors and do not necessarily represent those of their affiliated organizations, or those of the publisher, the editors and the reviewers. Any product that may be evaluated in this article, or claim that may be made by its manufacturer, is not guaranteed or endorsed by the publisher.
